# New software for statistical analysis of Cambridge Structural Database data

**DOI:** 10.1107/S0021889811014622

**Published:** 2011-06-08

**Authors:** Richard A. Sykes, Patrick McCabe, Frank H. Allen, Gary M. Battle, Ian J. Bruno, Peter A. Wood

**Affiliations:** a Cambridge Crystallographic Data Centre, 12 Union Road, Cambridge CB2 1EZ, UK

**Keywords:** data analysis, computer programs, Cambridge Structural Database, substructure, *Vista*

## Abstract

A collection of new software tools is presented for the analysis of geometrical, chemical and crystallographic data from the Cambridge Structural Database (CSD). This software supersedes the program *Vista*. The new functionality is integrated into the program *Mercury* in order to provide statistical, charting and plotting options alongside three-dimensional structural visualization and analysis. The integration also permits immediate access to other information about specific CSD entries through the *Mercury* framework, a common requirement in CSD data analyses. In addition, the new software includes a range of more advanced features focused towards structural analysis such as principal components analysis, cone-angle correction in hydrogen-bond analyses and the ability to deal with topological symmetry that may be exhibited in molecular search fragments.

## Abstract 2

A new piece of software for statistical analysis of geometrical, chemical and crystallographic data within the Cambridge Structural Database System is described. This software has been written specifically to deal with chemical structure data and crucially provides simultaneous visualization of the three-dimensional structural information.

## 1. Introduction

The Cambridge Structural Database (CSD; Allen, 2002 ▶) is the international standard repository for small-molecule crystal structures and is curated by the Cambridge Crystallographic Data Centre (CCDC). There are now more than 500 000 structures archived in the CSD (http://www.ccdc.cam.ac.uk/500000.php). This represents an enormous volume of information relating to intramolecular, intermolecular and crystallographic parameters. The CSD System incorporates an extensive suite of user-friendly and flexible tools for searching and analysing this wealth of information. Chemical knowledge extracted from the CSD is applicable to many areas of the chemical and physical sciences, especially pharmaceutical drug discovery, materials design, and drug development and formulation.

The CSD is frequently used for statistical analysis of intramol­ecular and intermolecular geometric structural parameters as well as other data types such as space group, colour, morphology and unit-cell dimensions. The program *Vista* (CCDC, 1994 ▶) has been the main statistical analysis tool in the CSD System since its original development. Since then, a great deal of the CCDC code base and programs have been replaced with newer and more advanced software written in C++. There remains a need in the CSD System for this type of analysis tool which is specifically tailored towards dealing with information extracted from crystal structures, and thus we are now turning our attention to upgrading this area of the system. This paper describes a new set of functionality developed to supersede *Vista*.

## 2. Overview

As the software requirements of users and the CSD System itself have evolved over the past few decades, more emphasis has been placed on three-dimensional visualization of data and closer interactivity between CCDC data analysis tools. To provide a more flexible and extensible framework for statistical analysis of CSD data, a new set of tools has been developed. These tools incorporate and extend the functionality previously contained in *Vista* and provide a highly interactive interface in which the data spreadsheet, histograms, scatterplots and the three-dimensional visualizer are all inter-connected. New options in this software over and above the *Vista* capabilities include a facility to deal accurately and easily with cases of topological symmetry in the CSD search fragment, as well as improved functionality to deal with circular descriptors, *e.g.* torsion angles.

This new software has been implemented as a plug-in to the program *Mercury* (Macrae *et al.*, 2008 ▶) and represents a step towards centralizing all the functionality contained in the CSD System. The following provides an overview of the new functionality, which includes all of the options available in the original *Vista* program together with many new features.

## 3. Technical details

### 3.1. Program language and architecture

The new tools are written in C++, as is *Mercury* itself, and use functionality provided by the CCDC’s C++ Toolkit (Bruno *et al.*, 2002 ▶). This toolkit is central to a large number of the programs now produced by the CCDC, including *Mercury* (Macrae *et al.*, 2008 ▶), *Mogul* (Bruno *et al.*, 2004 ▶), *IsoStar* (Bruno *et al.*, 1997 ▶), *WebCSD* (Thomas *et al.*, 2010 ▶) and *enCIFer* (Allen *et al.*, 2004 ▶).

### 3.2. Database back-end

When numerical data are transferred to *Mercury* for statistical analysis either from a *ConQuest* search, a *Materials* module packing feature search (Macrae *et al.*, 2008 ▶) or a raw data file, the information is stored in a relational database system (currently SQLite; http://www.sqlite.org) for fast access. This relational database system, packaged along with the CSD System, runs in the background and serves to ensure that look-up of data and interaction between plots and spreadsheets in the program is extremely fast.

## 4. Graphical user interface and visualization capabilities

### 4.1. Data sets and selections

Any data set read into the program will be displayed in a spreadsheet, allowing a range of options for browsing and sorting based on the columns or parameter descriptors that are available. New data sets introduced within a session are presented in separate spreadsheets, allowing the user to switch back and forward easily between data sets. Control can be applied over the individual data items shown at any one time using the concept of hidden data. This means that the user can quickly narrow down a data set to only display those data within a user-defined set of criteria.

**Figure 1:**
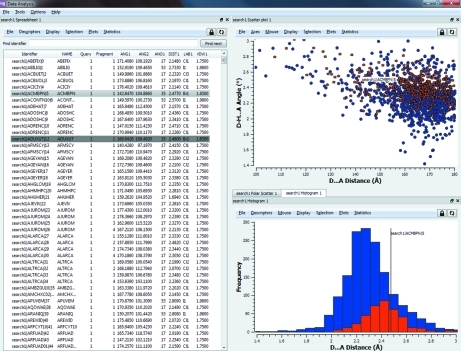
Illustration of the data analysis interface including the spreadsheet and two plot types. The data shown pertain to hydrogen bonds to halide ions and the selected points are those where the specific halogen involved is bromine.

To manipulate the data for analysis, selections can be made by using click and drag on the spreadsheet or in any of the plot windows. In addition, there are a number of further options for making selections, such as filtering, whereby cut-off criteria can be applied for each of the descriptors, and grouping, which allows simple grouping based on integer descriptors. Fig. 1 ▶ shows the data analysis software interface with a set of data points selected. The selection is shown across the scatterplot, histogram, spreadsheet and three-dimensional visualizer simultaneously and updates dynamically with any changes made to the selection.

### 4.2. Structure visualization

There are many data analysis tools and structural visualizers available, but this software provides those capabilities together in an integrated system. As the functionality described in this paper has been implemented using the CCDC Toolkit and is used by *Mercury* as a plug-in, it is very easy to visualize the specific data and parameters in the three-dimensional structures alongside the statistical and plotting features. Any selections made within the data analysis plots and tables (as described above) will be shown immediately within the *Mercury* structure visualizer. If a group of structures has been selected in the spreadsheet, these can also be simply browsed.

### 4.3. Plotting and charting

**Figure 2:**
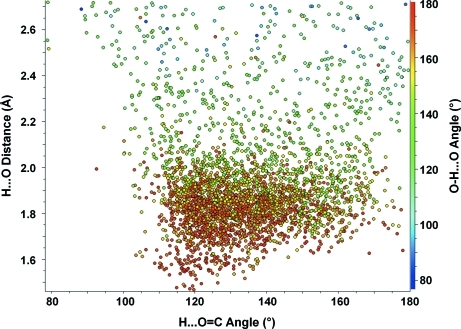
Scatterplot of H⋯O distance (Å) against H⋯O=C angle (°) for alcohol to ketone hydrogen bonds with O—H⋯O angle (°) shown using a colour scale.

A full range of charting and plotting options is available within the new statistical analysis tools, including histograms, polar histograms, Cartesian scatterplots, polar scatterplots and heat maps. It is also possible to indicate the variation in a third variable on scatterplots and polar scatterplots by colouring the symbols on a heat scale according to the values of this additional variable. This is illustrated in Fig. 2 ▶, showing a scatterplot of the hydrogen to acceptor distance (H⋯O) against the angle at the acceptor (H⋯O=C) in alcohol to ketone hydrogen bonds. The heat scale is used in this case to show the hydrogen-bond angle at the donor (O—H⋯O) as the third variable.

This plot shows clearly that the shorter hydrogen bonds observed in the CSD tend to have an angle at the acceptor of around 125–130° and an angle at the donor H atom close to 180°. Longer, and by implication weaker, hydrogen bonds are seen to have a greater spread in angle both at the acceptor and at the donor H atom.

### 4.4. Exporting images

Any of the charts or plots generated using these data analysis tools can be exported as an image for reference or publication purposes. Various configuration options are available for each of the graphing objects, including changing background colours, axis labels, symbol types and so on.

## 5. Numerical and statistical capabilities

### 5.1. Standard and circular descriptive statistics

The software will calculate a variety of statistical descriptors for a given distribution. These include the calculation of the mean, the variance, the standard deviation of the mean, the median and quantile values, as well as skewness and kurtosis amongst other measures. With these features it is therefore simple, for example, to quantify the spread or the asymmetry of the distribution.

When dealing with data of a circular or periodic nature, such as torsion angles, the distinction between high and low values is arbitrary and the designation of the zero position can vary according to some external convention. By chemical convention, torsion angles are generally measured on a range from −180° through 0° to +180°. The arithmetic averaging of torsions is therefore clearly problematic: at the simplest level a torsion angle with a unimodal distribution centred on 180° will produce a mean close to 0° when using this range, which is obviously absurd. In order to treat periodic variables correctly we need to apply the appropriate statistical model, that of circular statistics, which generates its own specialist descriptors of periodic distributions. This functionality is implemented within the software using methods as defined by Berens (2009 ▶). Users can choose to determine descriptive statistics for any descriptors based on regular statistics or treat them with circular statistics.

### 5.2. Principal components analysis

The method of principal components analysis (PCA; Chatfield & Collins, 1980 ▶) is based on transforming a set of potentially correlated variables into a new, and smaller, set of uncorrelated and mutually orthogonal variables termed principal components. This process can make it easier to understand multivariate data and can significantly aid the location and identification of clusters of observations having similar values. The technique is often used when trying to analyse the variation in a number of correlated molecular or intermolecular geometric parameters within a data set of related crystal structures. PCA has been used in combination with data mining to study, for example, clustering of molecular conformations (Perez *et al.*, 2002 ▶), the effect of hydrogen bonding on molecular geometry (Krygowski *et al.*, 2004 ▶) and metal coordination environments (Allen *et al.*, 2003 ▶).

**Figure 3:**
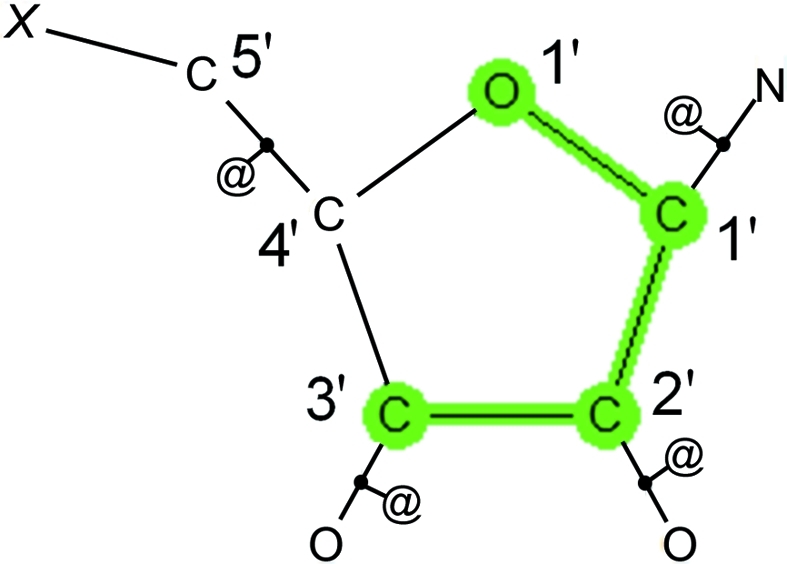
Query defined for aminofuranoside substructures. *X* refers to any atom and the @ symbol defines bonds as being acyclic.

We illustrate the value of PCA by investigating the intramolecular geometry of aminofuranoside rings in the CSD using a classic previous research study (Murray-Rust & Motherwell, 1978 ▶) as a basis. The raw data relating to aminofuranoside rings were extracted from the CSD by performing a search in *ConQuest* (Bruno *et al.*, 2002 ▶). The query was drawn as shown in Fig. 3 ▶, with each of the five internal torsion angles in the ring defined as numerical parameters. Also shown in Fig. 3 ▶ is the standard numbering scheme for this type of ring. To ensure that only higher-quality organic structures were included the following secondary acceptance criteria were applied: (i) no crystallographic disorder, (ii) no covalent polymeric (*catena*) bonding, (iii) no residual errors following CSD validation procedures, (iv) determined using single-crystal techniques (no powder diffraction structures), (v) having reported *R* ≤ 0.075 and (vi) restricting the search to organic structures according to CSD definitions (Allen, 2002 ▶; Bruno *et al.*, 2002 ▶).

**Figure 4:**
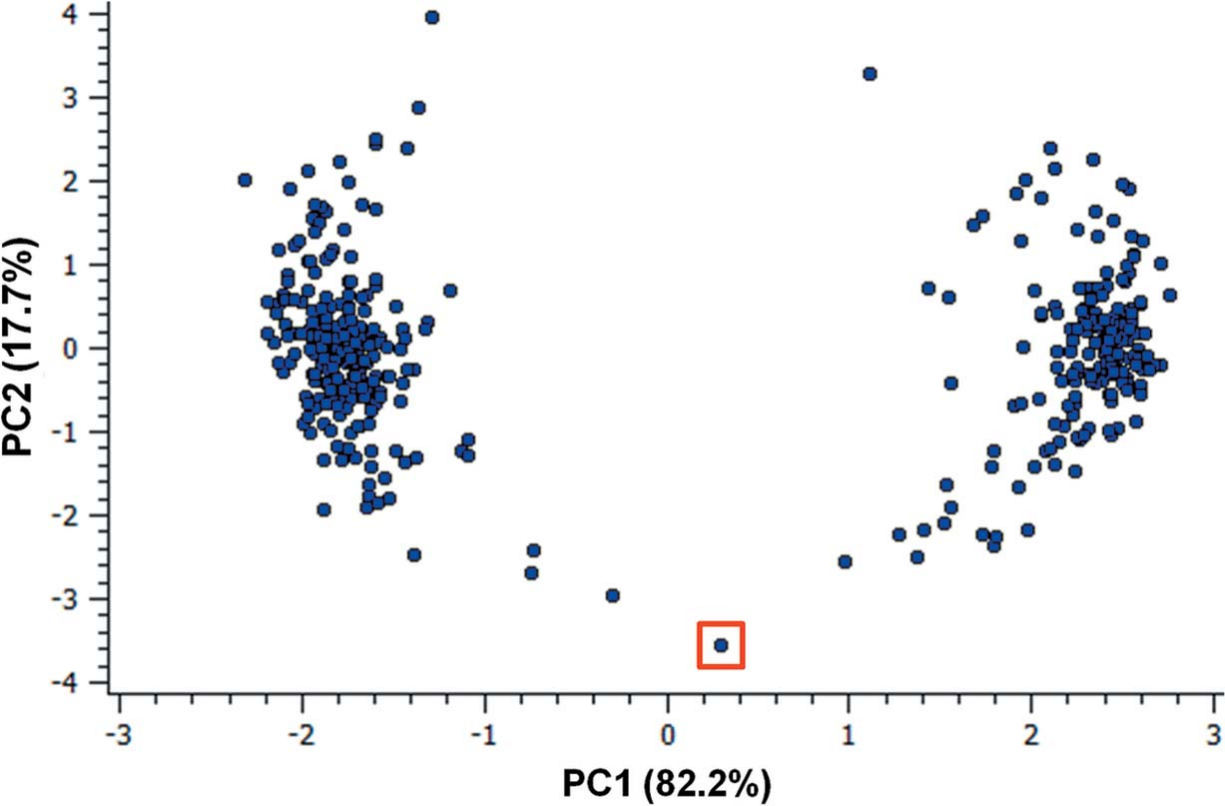
Scatterplot of the first two principal components relating to the conformation of aminofuranoside rings in the CSD. The percentage of the variance in the data explained by each principal component is shown in brackets. The outlier identified in the square box is discussed in the text.

The numerical results of this search could be analysed and plotted without modification, *e.g. via* pairwise torsional scatterplots. However, by applying PCA we hope to reduce significantly the number of parameters that describe the majority of the conformational variance in the data set. Analysis of the principal components indicates that just two components account for 99.99% of the variance in the data set. A scatterplot of these first two principal components is shown in Fig. 4 ▶.

**Figure 5:**
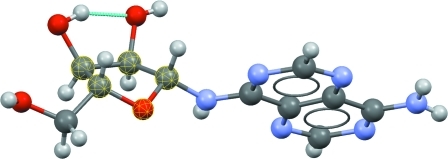
Three-dimensional molecular geometry in the structure of CSD refcode RPPYPY20, illustrating the unusual d-ribose ring conformation identified in Fig. 4 ▶.

The plot shows two clusters, which correspond to the two main ring conformational types present in the data set. The cluster on the left comprises C2′-*endo* rings, while those on the right are C3′-*endo*. Points towards the centre of the plot are representative of more unusual conformations, such as O1′-*endo*, and are indicative of possible pathways for ring deformation. If we select the point highlighted in the box in Fig. 4 ▶, for example, the corresponding structure will be shown immediately within the three-dimensional visualizer (Fig. 5 ▶).

This point corresponds to an O1′-*endo* ring fragment from the structure of 6-amino-10-(β-d-ribofuranosylamino)pyrimido[5,4-*d*]pyrimidine (CSD refcode RPPYPY20; Narayanan & Berman, 1975 ▶). It is clear from the PCA plot that this is indeed a very unusual d-ribose conformation. The authors of the structural paper note that this must generate a substantial strain energy, though they suggest this may be reduced by the intramolecular hydrogen bond formed between the O—H groups. This example illustrates how quickly it is possible to learn more about the conformational diversity of a given substructure using PCA, as well as the power of accessing numerical and three-dimensional visual data relating to the structures simultaneously – hyperlinking features that are not available in external statistical analysis software.

### 5.3. Topological symmetry

One of the most subtle issues connected with searching and analysing geometrical data in crystal structures, whether it is intra- or intermolecular information, is in dealing correctly with topological symmetry that may occur in the search fragment. Topological symmetry means that there are parameters in a structure that are chemically equivalent in the query, but are usually geometrically different in the crystal structure (unless the topological symmetry of the search fragment is coincident with a crystallographic symmetry element). Thus, a query comprising a phenyl ring has six chemically equivalent C—C—C angles, but these are typically geometrically independent in each crystal structure containing a phenyl ring.

This problem stems from the multiple ways of mapping the atoms and bonds of a topologically symmetric search fragment onto the atoms and bonds of each search hit in a crystal structure. This is discussed in some detail by Taylor & Allen (1994 ▶). The correct way to handle this problem is to define all of the parameters in the query (*e.g.* all six C—C—C angles in a phenyl ring), giving rise to multiple columns of data (six in the case of the phenyl ring angles) in the results spreadsheet. As the data in these columns represent chemically equivalent fragments, they should be treated as a single distribution.

**Figure 6:**
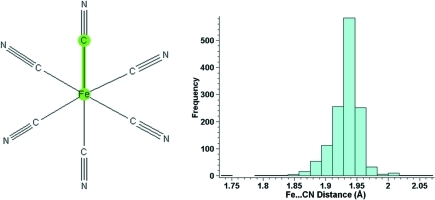
Query (left) and histogram of results (right) for Fe—CN distances in the CSD, illustrating the occurrence of query topological symmetry.

To make such analyses easier, a specific tool is now available in the software for combining multiple parameters and treating them as a single distribution for all plotting and analysis functions. The user has the ability to identify which columns should be treated as equivalent within the program. Fig. 6 ▶ shows an example of a search for iron cyanide complexes with bond distances measured between the metal and ligand atoms.

The query shown in Fig. 6 ▶ (left) has sixfold topological symmetry; however, it is unlikely that all the actual iron cyanide complexes observed in the CSD will exhibit this sixfold symmetry within their crystal structures. To ensure that each crystallographically independent bond distance is captured we define all six Fe—C bond lengths in the query and then combine the resulting data into a single distribution to give the histogram shown in Fig. 6 ▶ (right).

### 5.4. Cone-angle correction

When analysing angles involved in intermolecular interactions, such as *D*—H⋯*A* angles (θ) in hydrogen bonds, it is important to be aware of the difference between Cartesian and spherical polar coordinates. To generate an unbiased histogram showing the density of contacts based on a given parameter any bin of the histogram should, in principle, correspond to an equal volume in three-dimensional space.

**Figure 7:**
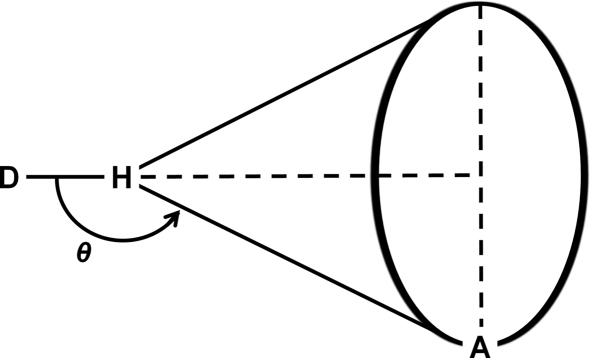
Hydrogen-bonding cone of approach for a given angle θ, where *D* represents a donor atom and *A* represents an acceptor atom.

When a hydrogen-bond donor (*D*—H) approaches an acceptor (*A*) there is essentially only one orientation of the donor that achieves a *D*—H⋯*A* angle of exactly 180°. The acceptor group sweeps out a possible cone of approach, which gets progressively larger as the interaction deviates further from linearity and therefore the θ angle decreases (see Fig. 7 ▶). This means that if a straightforward histogram of intermolecular *D*—H⋯*A* hydrogen-bonding angles is plotted, using bins of equal size, the distribution is inherently biased away from 180°. The number of feasible orientations for any value of θ is in fact proportional to sinθ by inspection of Fig. 7 ▶.

Kroon & Kanters (1975 ▶) showed that this effect was very noticeable for hydrogen bonds. Medium-strength hydrogen bonds that were believed to prefer linearity, such as neutral O—H⋯O interactions, when analysed *en masse* were seen to have a histogram maximum in the region of 165°. Their research indicated that by simply dividing the bin frequencies by sinθ the histogram can be corrected to account for this bias. The facility to apply this cone-angle correction is provided for the user within the histogram plotting options.

**Figure 8:**
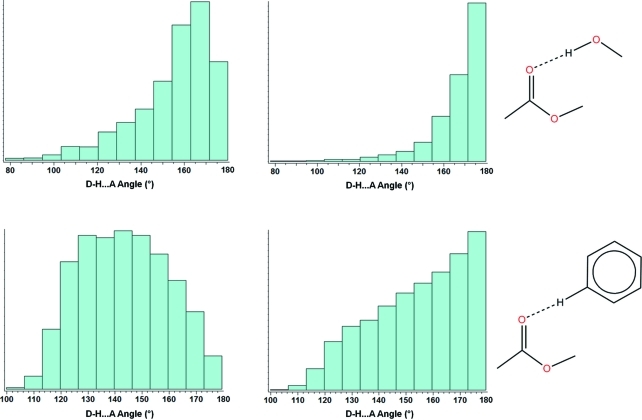
Unmodified (left) and cone-angle-corrected (right) histograms for the CSD distributions of *D*—H⋯*A* angles in hydrogen bonds to esters from (top) alcohol O—H and (bottom) phenyl C—H groups.

Fig. 8 ▶ shows unmodified histograms (left) and cone-angle-corrected plots (right) for the CSD distributions of *D*—H⋯*A* angles in two different hydrogen-bonding interactions. In each case the acceptor group is an ester but the donor group differs based on donor strength, with alcohol O—H (top) being a strong donor and phenyl C—H (bottom) being a substantially weaker donor.

It is clear from the unmodified histograms that the peak in the CSD distribution is shifted substantially away from linear, *i.e.* 180°. In the case of the strong and highly directional hydrogen bond involving an alcohol donor, the peak is at roughly 160°. After correcting the histograms to account for the cone-angle geometrical bias we can see that both interactions do actually prefer to be linear. In fact, a recent study has shown that even very weak hydrogen bonds have a strong energetic preference for linearity about the donor H atom (Wood *et al.*, 2009 ▶). The stronger hydrogen bond (alcohol to ester) does, however, show a distribution more tightly clustered around 180° compared to the phenyl interaction; this highlights once more the link between interaction strength and hydrogen-bond linearity.

### 5.5. Correlation, covariance and significance

For further analysis of multivariate data sets, the ability to determine correlations and covariances between descriptors has also been included in the software. Here you can calculate correlations, Spearman rank correlations and covariances for any number of descriptor pairs. These options essentially provide information about the relative dependence between descriptors.

A fixed-level hypothesis test is also provided. Given a selection in the data set, at the significance level specified by the user, the following hypotheses are tested using Student’s two-sample *t*-test: 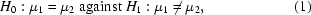
where μ_1_ represents the mean parameter of the selected items and μ_2_ represents the mean parameter of the non-selected items. Tests resulting in a rejection of the null hypothesis (*H*_0_) are highlighted in a configurable colour. The significance probability (*p* value) is also calculated and reported, which allows the user to make their own assessment of the weight of evidence against the null hypothesis.

As an example we can return to the selection shown in Fig. 1 ▶, relating specifically to bromide acceptors within a set of hydrogen bonds to halide ions. We find in this case that the mean *D*—H⋯*A* angle to bromides is different from the mean hydrogen-bonding angle to the other halides at the 1% significance level. The significance probability (0.003) confirms that there is strong evidence against *H*_0_.

## 6. Documentation, availability and environment

The new data analysis features within *Mercury* are fully documented and there are several tutorials available to illustrate their use. Documentation can be accessed through the program interface or *via* the CCDC web site (http://www.ccdc.cam.ac.uk/). This functionality is accessible within *Mercury* and is available to all users with a registered copy of the Cambridge Structural Database System. The software described in this paper is supported on a range of platforms including Windows (Intel compatible, 32 bit: Windows XP/Vista/7), Linux (Intel compatible, 32 bit: Red Hat Enterprise 3, 4, 5; SUSE 10, 11; Debian 4.0, 5.0) and Mac OSX (10.4, 10.5, 10.6).
